# Verification of Type-A and Type-B-HC Blinking Mechanisms of Organic–Inorganic Formamidinium Lead Halide Perovskite Quantum Dots by FLID Measurements

**DOI:** 10.1038/s41598-020-58926-3

**Published:** 2020-02-07

**Authors:** Cong Tai Trinh, Duong Nguyen Minh, Kwang Jun Ahn, Youngjong Kang, Kwang-Geol Lee

**Affiliations:** 1 Department of Physics, Hanyang University, Seoul, 04763 Republic of Korea; 2 Department of Chemistry, Research Institute for Natural Sciences, Institute of Nano Science and Technology, Hanyang University, Seoul, 04763 Republic of Korea; 3 Department of Physics and Department of Energy Systems Research, Ajou University, Suwon, 16499 Republic of Korea

**Keywords:** Atomic and molecular physics, Optical physics, Physical chemistry

## Abstract

Organic–inorganic halide perovskite nanocrystals or quantum dots (PQDs) are excellent candidates for optoelectronic applications, such as lasers, solar cells, light emitting diodes, and single photon sources. However, the potential applications of PQDs can expand once the photoluminescence, and in particular, the blinking behaviors of single PQDs are understood. Although the blinking of PQDs has been studied extensively recently, the underlying mechanism of the blinking behaviors is still under debate. In this study, we confirmed that type-A and type-B-HC (hot carrier) blinking, contributed to PQD blinking using their fluorescence lifetime intensity distribution (FLID). Type-B-HC blinking was experimentally confirmed for the first time for formamidinium based PQDs, and the simultaneous contributions of type-A and type-B blinking were clearly specified. Further, we related different FLID data to the ON/OFF time distribution as distinct features of different blinking types. We also emphasized that detection capability was crucial for correctly elucidating the blinking mechanism.

## Introduction

Lead halide perovskites quantum dots (PQDs) have been successfully used as materials for next-generation optoelectronic devices, such as solar cells, light emitting diodes, lasers, and single photon sources^1–7^. Their primary advantages lie in their large absorption cross-section, high photoluminescence (PL) quantum yield, narrow PL spectral width, wide-range emission tunability, feasibility for scale-up synthesis, and cost-effectiveness^8–12^. Despite their advantages, blinking and irreversible photo-degradation of PQDs have been frequently reported and could limit their optical performance^13,14^.

Classifying blinking scenarios into type-A and type-B has been widely accepted. Type-A blinking mechanism is attributed to charging and discharging processes. When quantum dots (QDs) are charged owing to carrier transfer to trapped states, the non-radiative Auger recombination of the trion state competes with the radiative recombination to quench photon emission by transferring its exciton energy to the extra carrier (electron or hole), which results in low PL emission (OFF state). The high PL emission (ON state) is recovered by neutralizing the QDs^15–17^. Type-B blinking is attributed to the activation and deactivation of multiple recombination centers (MRCs)^17–19^, which are short-lived traps (*e.g*., shallow traps) in QDs^17–20^. The activation and deactivation of MRCs modulate the non-radiative recombination rates, and thus, cause PL intensity fluctuations^20^. These two types of blinking mechanisms could be used for conventional semiconductor QDs^15–20^ and also, possibly, for PQDs^13,21–23^. In addition, several researchers have suggested in their recent publications, that the two types of blinking simultaneously contribute to PQD blinking^24–27^.

Analysis of fluorescence lifetime intensity distribution (FLID) has been regarded as a very effective experimental approach to distinguish between these two types of blinking mechanisms^18,19^. While the FLID trajectory for type-A blinking is typically a curved line^18,19^, that of type-B blinking can be further divided into two sub categories: band-edge carrier trapping (type-B-BC) which yields a linear relationship between the PL intensity and lifetime^19^, and hot-carrier trapping (type-B-HC) where the lifetime is almost constant irrespective of the large changes in PL intensity^18^.

Several scholars identified type-A blinking for PQDs using the FLID characteristics and assigned the observed short lifetimes to the trion^13,21,24^. However, the lifetimes of the PQD trion states have been reported to be vastly different, such as 170 ps^21^, 410 ps^28^, 2 ns^24,25^, and 5.8 ns^29^. These values could be comparable with the low-emission state lifetime in type B-BC blinking^23–25^. Moreover, the FLID trajectories of type-A blinking of PQDs in previous studies were not clearly verified compared to the conventional (cadmium chalcogenides) QD. In addition, owing to their similar shapes, the FLID trajectories of type-A and type-B-BC blinking of PQDs have not been clearly distinguished from each other. Therefore, it would be meaningful to further check the validity of recently given explanations on the FLID studies of PQDs.

In this study, we verified that two types of blinking mechanisms, type-A and type-B-HC, contribute for organic–inorganic formamidinium lead halide (FAPbBr_3_) PQDs^26^. This was accomplished by measuring the FLID and using the ON/OFF time distribution analysis from our previous study^26^. A clear curved line for type-A blinking and no changes in lifetime for type-B-HC blinking were clearly observed. Interestingly, for several single PQDs, type-A and type-B-HC blinking appeared simultaneously. We also analyzed the relationship between the FLID histogram and ON/OFF time distribution to further support our claims on the involved blinking mechanism. In addition, we found that the high detection efficiency and corresponding analysis (temporal) resolution would be crucial for correctly designating the blinking mechanism.

## Results and Discussion

Colloidal FAPbBr_3_ PQDs were synthesized using the ligand assisted reprecipitation method utilizing PbBr_2_-dimethyl sulfoxide (DMSO) complexes as precursors at room temperature^30^. The average size of the FAPbBr_3_ QDs was 11 ± 3 nm. More details are presented in the Methods section.

The FAPbBr_3_ PQDs solution in toluene was diluted with 3 wt% poly (methyl methacrylate) (PMMA) solution before being spin-coated onto a clean cover glass substrate, which allowed to separate individual QDs. The FAPbBr_3_ PQD samples were excited using a pulsed laser at a wavelength of 380 nm (76 ps pulse width and 20 MHz repetition rate) utilizing an in-house built confocal microscope system. The emitted photons were collected using an objective lens (Plan Apo VC, Nikon, numerical aperture of 1.4) and detected using avalanche photodiodes (APDs, SPCM-AQ4C, Perkin Elmer). The data was recorded using the two-channeled time-tagged time-resolved (TTTR) mode of the PicoHarp 300 (PicoQuant) photon counter. Spatial filtering was used to select only signal from the regions near the QD positions, thus reducing spurious background noise. All measurements were performed at room temperature.

For each PQD, we performed the second order photon correlation function g^(2)^(τ) measurement to guarantee the single particle in the detection area (as can be seen in Supplementary Information (Fig. S1)). Then, the obtained TTTR data were analyzed using a customized algorithm coded using the MATLAB software. For each bin time, the average lifetime was calculated by taking a weighted average of the decay time histogram^18,19^. Here, we note that the average lifetime can be inaccurately derived with limited pump-pulse repetition rate of 20 MHz. In Supplementary Information (Figs. S2 and S3), through a quantitative analysis, we conclude that this experimental limitation is not a critical problem for deciphering the FLID features for our target QDs.

**Figure 1:**
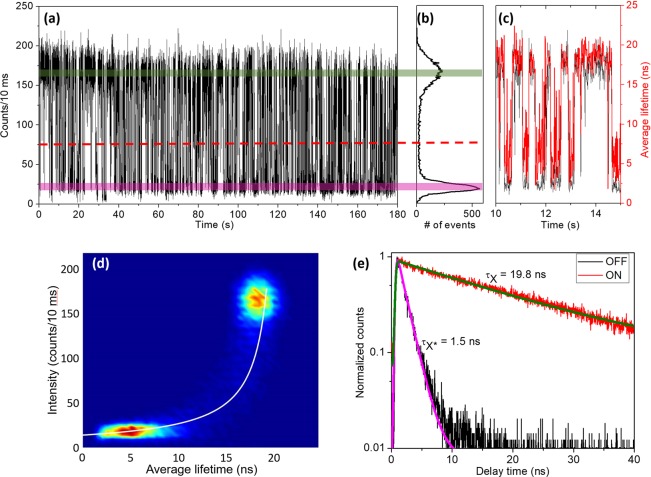
(**a**) PL intensity time trajectory at 10 ms bin time and (**b**) corresponding histogram for a selected perovskite quantum dot (PQD1). The green and pink colored areas indicate the ON and OFF sections, respectively, and the red dashed line is the threshold separating the ON and OFF states. (**c**) PL intensity and average lifetime (black and red lines, respectively) of selected small area in (**a**). (**d**) FLID histogram of PQD1. The white line is the FLID trajectory line fitted using Eq. (1). (**e**) Lifetime decay curves of the ON and OFF states selected in (**a**). Here, τ_X_ and τ_X*_ are the decay times of the single-exciton and trion, respectively.

Figure 1a presents the PL intensity time trace of a selected PQD (PQD1) recorded under pulse laser excitation. The excitation intensity was controlled to yield the average QD exciton number per pulse, <*N* > ~0.1^13^. The corresponding histogram is illustrated in Fig. 1b. A short interval of the PL intensity time trace (black line) from Fig. 1a is shown together with the average lifetime (red line) in Fig. 1c. The time bin size was 10 ms. A clear correlation between the PL intensity (ON/OFF) fluctuations and the lifetime can be observed. The FLID histogram of PQD1 is displayed in Fig. 1d. The lifetimes of the ON and OFF states were derived by selecting only the ON or OFF intensity sections (green and pink areas, respectively, in Fig. 1a,b) and fitting them using single exponential decay functions, as illustrated in Fig. 1e. The ON and OFF lifetimes were 19.8 and 1.5 ns, respectively.

The average lifetime was calculated based on the average photon arrival time relative to the laser pulse. During the unity bin time, *T*, a single PQD could remain in state 1 (*e.g*., ON state, the corresponding intensity and lifetime being *I*_1_ and *τ*_1_, respectively) or in state 2 (OFF state, the corresponding intensity and lifetime being *I*_2_ and *τ*_2_, respectively). The PQD could also remain in state 1 for the *T*_1_ (a fraction of *T*) and in state 2 for the rest of the time (*T*_2_ = *T − T*_1_). Then, the average intensity and lifetime, *I* and *τ*, respectively, for time *T* could be calculated as follows^19^:

$I=\frac{{I}_{1}{T}_{1}+{I}_{2}{T}_{2}}{T}$ and $\tau =\frac{{I}_{1}{T}_{1}{\tau }_{1}+{I}_{2}{T}_{2}{\tau }_{2}}{{I}_{1}{T}_{1}+{I}_{2}{T}_{2}}$. Thus, *I* is inversely proportional to *τ*:

1 $$I=\frac{{I}_{1}{I}_{2}({\tau }_{2}-{\tau }_{1})}{{I}_{2}({\tau }_{2}-\tau )+{I}_{1}(-{\tau }_{1}+\tau )}.$$

The PL intensity as a function of average lifetime, obtained by using Eq. (1) with experimentally obtained values, is plotted as a white solid line in Fig. 1d. Note that no fitting parameters were used. The curvature of the line in Fig. 1d is clearly different from the linear lifetime–intensity correlation for type-B-BC blinking, which assures that type-A blinking was responsible for the blinking mechanism of PQD1.

**Figure 2:**
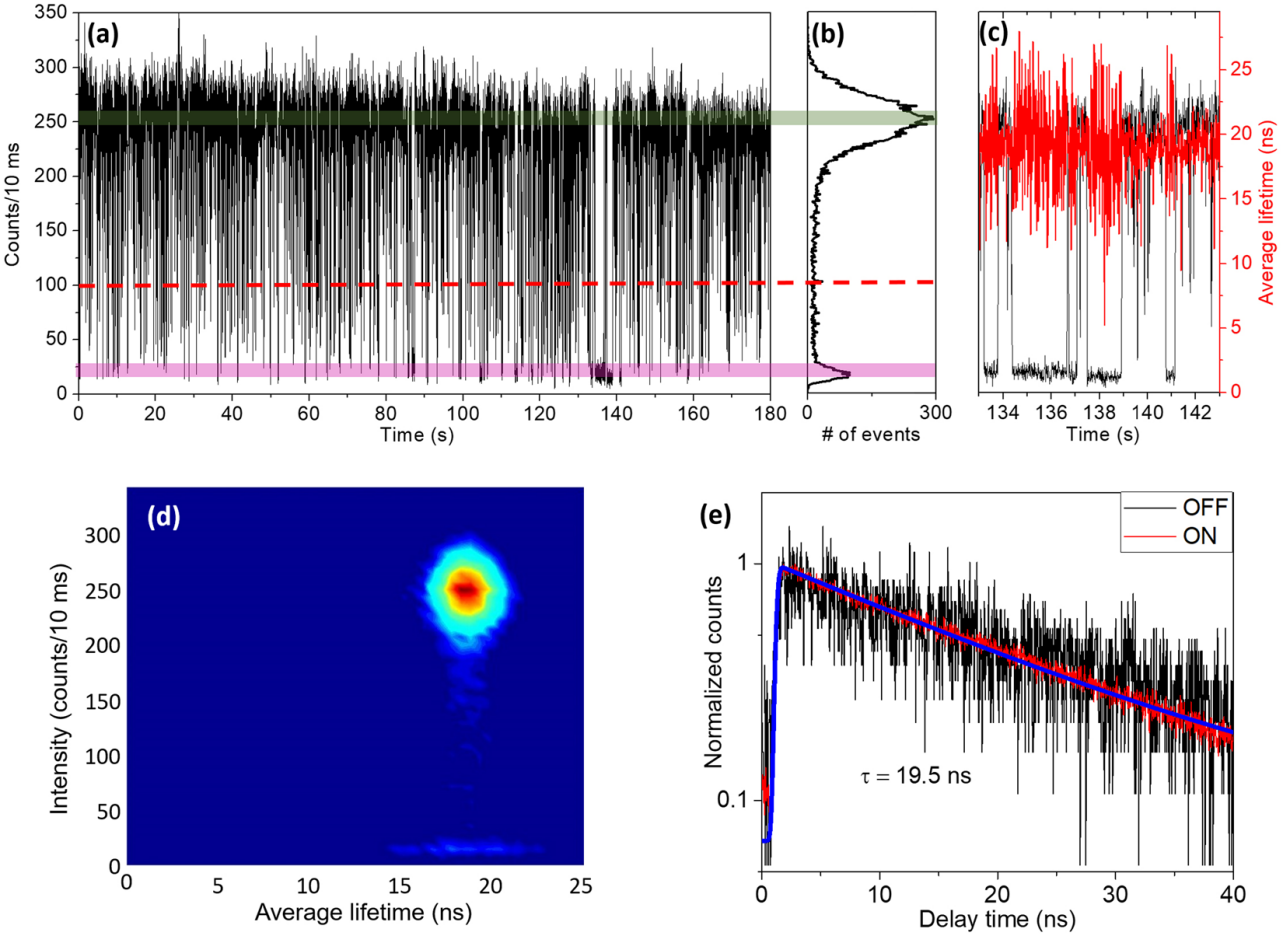
(**a**) PL intensity trajectory at 10 ms bin time and (**b**) corresponding histogram for a selected perovskite quantum dot (PQD2). The green and pink areas indicate the ON and OFF sections, respectively, and the red dashed line is the threshold that distinguishes the ON and OFF states. (**c**) PL intensity and average lifetime (black and red lines, respectively) of selected small area in (**a**). (**d**) FLID histogram of PQD2. (**e**) Decay curves of ON and OFF states selected in (**a**); here *τ* is the average lifetime.

Figure 2a,b present the PL intensity time trace and corresponding histogram of a different PQD (PQD2) at a similar excitation level, <*N*> ~0.1. The applied bin time was 10 ms as in Fig. 1. While the intensity trace in Fig. 2c clearly displays the ON and OFF fluctuations, the average lifetime does not present any clear correlation with the PL intensity. This feature can also be clearly observed in the FLID histogram in Fig. 2d. Figure 2e displays the decay curves of the green and pink regions in Fig. 2a,b, which correspond to the ON and OFF regions, respectively. A single exponential decay fitting yields lifetime of 19.5 ns (blue line). This behavior could be attributed to type-B-HC blinking where hot electrons are captured by activated non-radiative recombination centers (RC) before they relax into lower energy states^18^. To the best of our knowledge, this would be the first report on type-B-HC blinking observed in formamidinium based PQDs.

**Figure 3:**
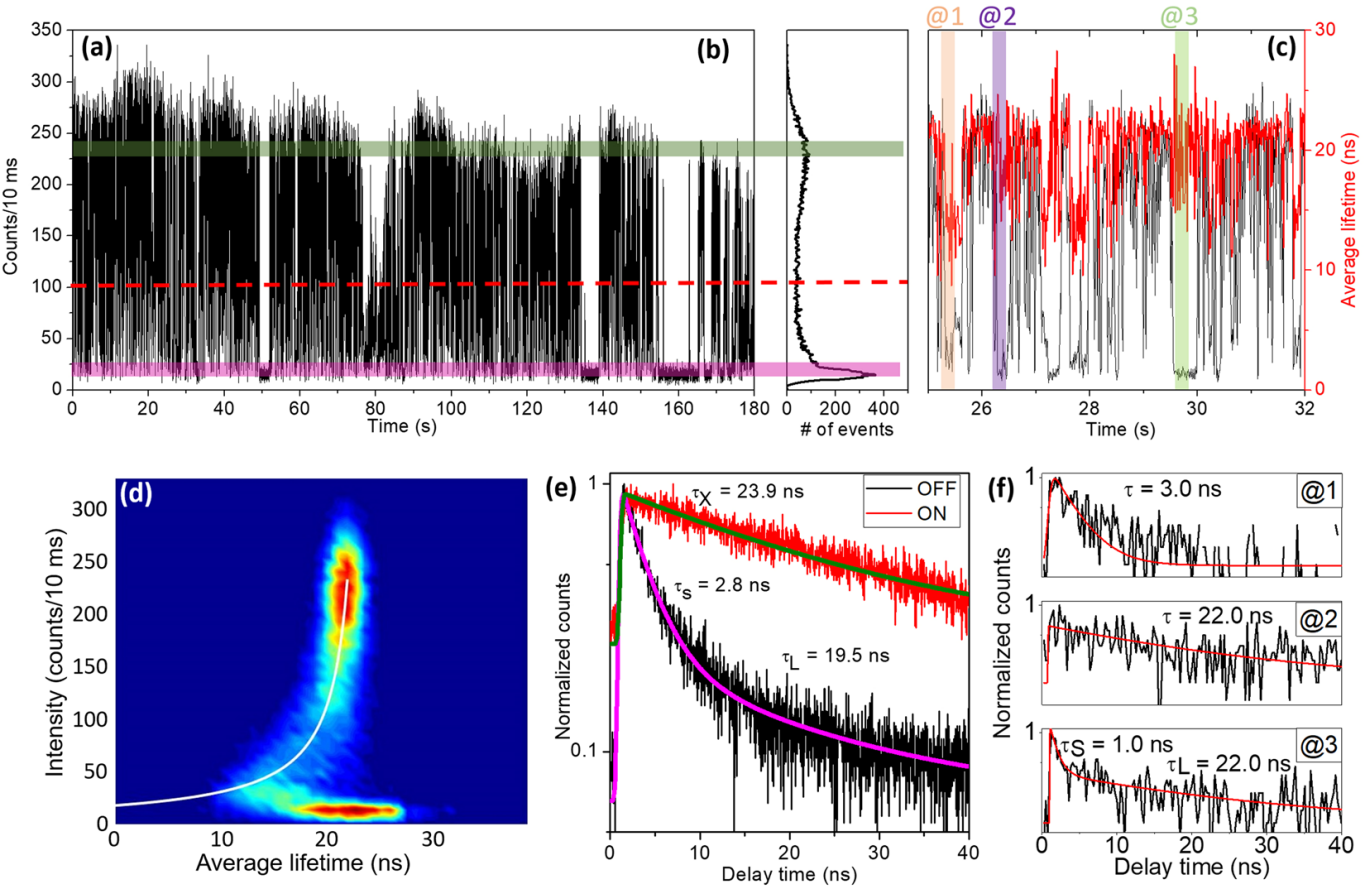
(**a**) PL intensity trajectory at 10 ms bin time and (**b**) corresponding histogram for a selected perovskite quantum dot (PQD3). The green and pink areas indicate the ON and OFF sections, respectively, and the red dashed line is the threshold that distinguishes the ON and OFF states. (**c**) PL intensity and average lifetime (black and red lines, respectively) of selected small area in (**a**). Three short OFF intervals are colored and marked as @1–3. Their corresponding lifetimes are compared in (**f**). (**d**) FLID histogram of PQD3. The white line is the fitted FLID trajectory line. (**e**) Decay curves of selected ON and OFF states in (**a**). τ_X_ is single exciton lifetime, τ_S_ and τ_L_ are fast- and slow-component of decay time of the OFF state. (**f**) Decay curves of selected regions in (**c**).

Pure type-B-HC blinking occurred rarely in our measurements (2 PQDs out of 25). In most cases, pure type-A blinking (13 PQDs out of 25) or combined type-A and type-B-HC blinking (10 PQDs out of 25) was observed. More FLID histograms can be found in Supplementary Information (Figs. S4–S6). Figure 3a,b present the PL intensity time trace and corresponding histogram of a different PQD (PQD3) at an excitation level of <*N*> ~0.1. The simultaneous contributions of type-A and type-B-HC blinking obtained for PQD3 are demonstrated in the FLID histogram of Fig. 3d. While the white curved line indicates type-A blinking, the quasi-constant lifetime in the OFF state is a characteristic of type-B-HC blinking^18^. It should be noted that the relatively large lifetime distribution of the OFF state was caused by overestimating the lifetime due to the flat background when the PL counts were close to the noise level^18,19^. In addition, the dark count effect could lead to considerable errors for the average lifetime estimation^18^. Therefore, to further support our claim of the coexistence of the two types of blinking, we presented in Fig. 3e the lifetimes of the ON and OFF states (green and pink regions, respectively, in Fig. 3a). While the ON state exhibited single exponential distribution corresponding to the lifetime of 23.9 ns, the OFF state could be fitted using a bi-exponential function, which yielded the fast decay time of 2.8 ns followed by a slow component of 19.5 ns. This slow decay lifetime for the OFF state indicated the presence of type-B-HC blinking, where hot electrons might be captured by the RCs before their non-radiative recombination with holes^21^. In addition, Fig. 3f @1–3 present the PL decay curves during selected short OFF intervals in Fig. 3c, which are highlighted in light orange, purple, and green, respectively. The decay curves could be fitted using mono- or bi-exponentials. The short (1 and 3 ns) and long (22 ns) lifetimes at different OFF intervals were a strong evidence for the coexistence of type-A and type-B-HC blinking in this PQD^18^. We did not observe any type-B-BC blinking for the FAPbBr_3_ PQDs under investigation. This may have occurred because the hot electrons relaxed much faster to RCs than to band-edges. It is interesting to note that among caesium (Cs), methylammonium (MA) and formarmidinium (FA) based lead halide perovskites, type B-HC is found for MA-^27^ and FA-based perovskite (this work) but not for Cs-based perovskites^13,22–25,28^. These results may suggest the different influence between organic (FA, MA) and inorganic cation (Cs) to PQD blinking behaviors. More studies are necessary to elucidate this point.

**Figure 4:**
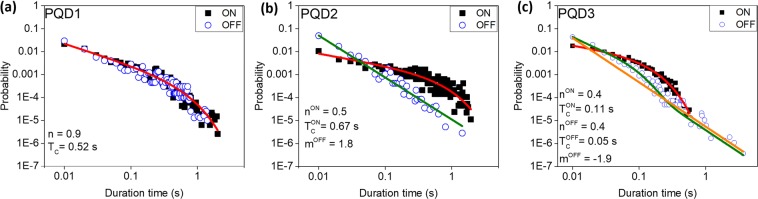
ON/OFF probability distribution of single PQDs (**a**) PQD1, (**b**) PQD2, and (**c**) PQD3.

The analysis of the ON/OFF time distribution in the PL intensity time trajectory could also be used to determine the blinking mechanism. In brief, the threshold intensity was selected, and intensities above and below the threshold were considered to represent ON and OFF states, respectively. We calculated the probability distributions, *P*(*τ*_ON/OFF_), for the ON/OFF periods, *τ*_ON/OFF_, as follows:

$$P({\tau }_{ON/OFF})=\frac{N({\tau }_{ON/OFF})}{{N}_{ON/OFF}^{tot}}\frac{1}{\delta {\tau }_{ON/OFF}},$$

where the number of occurrences of a given event, $N({\tau }_{ON/OFF})$, was divided by the total number of events, ${N}_{ON/OFF}^{tot}$, and average time duration between neighboring pre- and post-events, $\delta {\tau }_{ON/OFF}$^31^. The ON/OFF time distributions of PQDs 1–3 are illustrated in Fig. 4a–c, respectively. The ON time could be fitted using the power law with exponential cut-off $(\,\sim \,{\tau }^{-n}\exp (\,-\,\frac{\tau }{{T}_{C}})$, where *n* is the power law exponent following cut-off time *T*_*c*_) in all cases. However, the OFF time distribution was different for different types of blinking. For PQD1, the OFF time distribution could be fitted using the same function used to fit the ON distribution for type-A blinking. For PQD2, the OFF distribution could be fitted using a pure power law (~*τ*^−*m*^), which supported the occurrence of type-B-HC blinking. These results were also consistent with the classification introduced by Galland *et al*.^18^. For PQD3, instead of pure power law (orange solid line in Fig. 4c), the OFF time distribution could be well fitted using the following function (green solid curve in Fig. 4c):

2 $$P(\tau )={C}_{0}{\tau }^{-n}\exp (\,-\,\frac{\tau }{{T}_{C}})+{D}_{0}{\tau }^{-m}$$

which was consistent with our previous study^26^ and supported our suggestion of the simultaneous contributions of two types of blinking in PQDs.

Herein, we stress the importance of measurement capability and time-resolution during blinking analysis. The different bin times used when analyzing the PL intensity trajectories could change the results quite significantly^32,33^, thus the cut-off behavior can be easily hidden into pure power law distribution. In this study, we applied two different photon measurement schemes: TTTR T3 and T2 modes. The T3 mode used one input channel as excitation timing reference synchronized to the pump pulses. Therefore, the other input channel counted the signal photons emitted by PQDs. In addition, the acquisition timing resolution was limited to 1 ns. By comparison, the T2 mode used both input channels to count the signal photons, and the timing resolution was higher: 4 ps^34^. Therefore, the larger detection photon counts and higher timing resolution of the T2 mode could allow for a smaller bin size when analyzing the PL intensity trajectory. However, lifetime analysis was only possible for the T3 mode.

**Figure 5:**
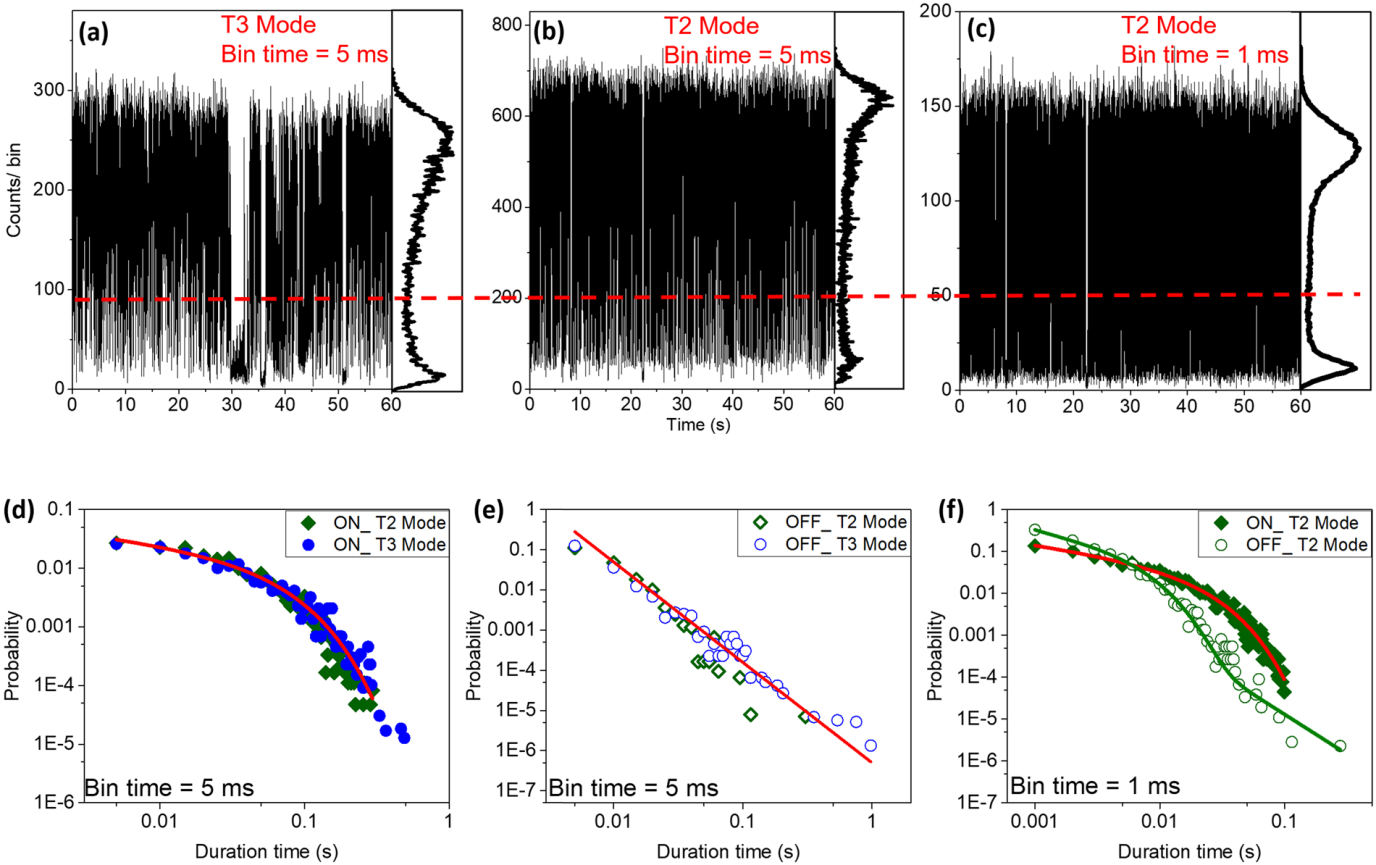
PL trajectory and corresponding histogram recorded in (**a**) T3 and (**b**) T2 modes at 5 ms bin time for a selected perovskite quantum dot (PQD4). (**c**) Same data set of PDQ4, but recorded in T2 mode at 1 ms bin time. The red dashed line is the threshold that distinguishes the ON and OFF states. (**d**) ON and (**e**) OFF time distributions from PL trajectories in (**a**) and (**b**). (**f**) ON and OFF time distributions of PL trajectory in (**c**).

To quantitatively compare the two detection modes, we analyzed the PL intensity time trajectories of PQD4 under the same pulse laser illumination conditions but using different TTTR modes (T2 and T3). Figure 5a,b were obtained in the T3 and T2 modes, respectively, and were analyzed for the same bin time of 5 ms. The detection count of the T2 mode was roughly 2.5 times larger than that of the T3 mode. The ON time duration illustrated in Fig. 5d presents a power law distribution followed by cut-offs for both modes. The OFF time durations presented in Fig. 5e could be fitted using pure power laws (without cut-offs) for both modes, and the errors were acceptable. However, for the T2 mode, it was possible to further reduce the bin time with enough photon counts as shown in Fig. 5c. For the bin time of 1 ms, the ON time still presented a power law distribution with cut-off profile, but the OFF time exhibited mixed contributions of two types of blinking, as described by Eq. (2) (Fig. 5f). Our results indicate strongly that a shorter bin time maintaining higher photon detection capability is a crucial condition to precisely analyzing blinking behaviors of PQDs, especially when the cut-off time becomes comparable to binning time.

## Conclusions

In conclusion, we demonstrated that type-A and type-B-HC blinkings, solely or simultaneously, contributed to FAPbBr_3_ QDs blinking. This was accomplished by analyzing the FLID and ON and OFF duration times. The specific curvature of type-A blinking was clearly visualized, and the type-B-HC blinking was observed for the first time for formamidinium based PQDs. The simultaneous contributions of two different blinking types were verified using FLID and were further supported by the ON/OFF duration statistics data. This simultaneous blinking behavior was also observed for traditional colloidal CdSe/CdS core-shell QDs. This similarity suggests that it could be possible to control the blinking behavior of PQDs using pre-treatment methods, such as thick shell or Fermi-level modifications. In addition, we also suggest that the unusual shape of the OFF time distribution (which was described using Eq. (2)) could be a distinct feature that would allow to distinguish blinking types. However, attention should be paid to correctly reconstruct the OFF time distribution using sufficient time resolution.

## Methods

### Synthesis of FAPbBr_3_ QDs

The FAPbBr_3_ QDs analyzed in this study were synthesized following the procedure in a previous report^26,30^. Briefly, precursor solution was first prepared by mixing 0.1 mmol (0.0445 g) PbBr_2_-DMSO, 0.1 mmol (0.0125 g) formamidinium bromide, and 20 μL OLA in 7 mL anhydrous DMF. The precursor solution was then transferred dropwise into a large amount of toluene (175 mL)/OLAc (787 μL) mixture while stirring vigorously. Upon mixing with toluene, the solution immediately turned pale green. Lastly, some large particles were removed from the FAPbBr_3_ nanocrystal solution by centrifugation at 5300 RCF for 10 min.

### Preparation of thin film

The PMMA film embedding single FAPbBr_3_ QDs is prepared by spin coating (2000 rpm for 45 s). More experimental parameters can be found in ref. ^26^.

### Photon detection and analysis

When obtaining the PL intensity time traces and lifetimes, photons were detected using single photon counting APDs (SPCM-AQ4C, Perkin Elmer). TTTR T3 mode (1 ns resolution) is used for the lifetime measurements with using two channels of the PicoHarp 300 (PicoQuant). Both the T2 and T3 mode are used for the PL intensity time trace. All collected data were reconstructed and analyzed using algorithms, coded utilizing the MATLAB software, and fitted employing the ORIGIN 2016 software. The temporal resolution of the photon detection system used in this study was estimated to be approximately 0.65 ns by measuring temporally short laser pulses (~3 ps), and de-convoluting the measured curves.

## Supplementary information

Supplementary Information.
