# Supplementary material for: Verification of Type-A and Type-B-HC Blinking Mechanisms of Organic–Inorganic Formamidinium Lead Halide Perovskite Quantum Dots by FLID Measurements
Source: Sci Rep. 2020 Feb 7;10:2172. doi: 10.1038/s41598-020-58926-3 (PMC7005873; doi:10.1038/s41598-020-58926-3)
Supplement: Supplementary file 1 — Supplementary Information. [file 41598_2020_58926_MOESM1_ESM.pdf]

Supplementary Information for

Verification of Type-A and Type-B-HC Blinking Mechanisms of Organic–Inorganic Formamidinium Lead Halide Perovskite Quantum Dots by FLID Measurements

Cong Tai Trinh,[1] Duong Nguyen Minh,[2] Kwang Jun Ahn,[3] Youngjong Kang,[2] and Kwang-Geol Lee[1,*]

[1]Department of Physics, Hanyang University, Seoul 04763, Republic of Korea

[2]Department of Chemistry, Research Institute for Natural Sciences, Institute of Nano Science and Technology, Hanyang University, Seoul 04763, Republic of Korea

[3]Department of Physics and Department of Energy Systems Research, Ajou University, Suwon 16499, Republic of Korea

[*]Address correspondence to kglee@hanyang.ac.kr

S1. Second order photon correlation function $g^{(2)}(\tau)$

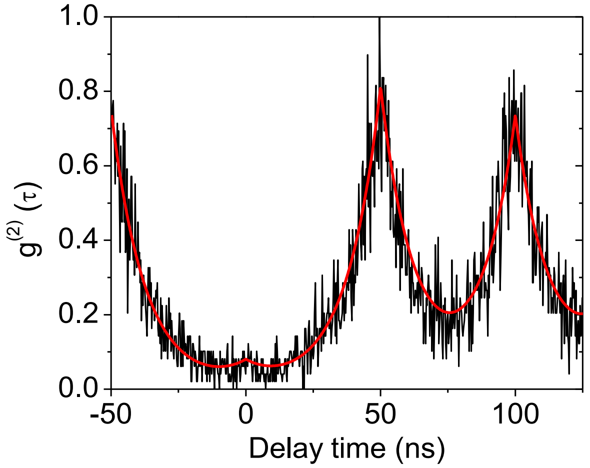

Figure S1. Second order photon correlation function $g^{(2)}(\tau)$ of a single perovskite quantum dot on a glass substrate.

S2. Effect of the limited pulse repetition rate (20 MHz) on the average lifetime

We carried out a quantitative analysis on the effect of the limited pump repetition rate on the measured lifetime. Here, we compared two different methods; the average arrival time $\bar{t}$ applied in our main manuscript and the decay time τ obtained by fitting the data with a single decaying exponential. In Figure S2(a), we assumed three single exponential decay curves with their lifetimes of 20 ns (black), 10 ns (red), and 5 ns (blue), respectively. Then the average arrival time $\bar{t}$ (calculated for 0 < delay time < 50 ns) is found to be smaller than τ by 22.1%, 4.3%, and 2.0%, respectively. While the error (the discrepancy between values obtained by two methods) for $\tau < 10$ ns is suitably small (< 5%), for $\tau > 10$ ns it becomes to be considerable. Because the real data is composed of multi-exponentials with different decay constants, we derived the error value from a real experimental data. For each bin time of 10 ms (one point in the FLID plot), we constructed the photon arrival time distribution (blue dots in Figure S2(b)), then obtained τ by a single exponential fitting and $\bar{t}$ by taking the weighted averaging method. We repeated the same processing for different points in the FLID plot and reconstructed the relations between τ and $\bar{t}$ as shown in Figure S2(c). Black data points and red dashed line represent $\bar{t}$ and τ, respectively. Here, we omitted the range of $\tau < 10$ ns. This is because, for a shorter lifetime, the distributional shape differs further from a single decaying exponential, therefore τ obtained from a single exponential fitting cannot be close to the real decay time anymore. Interestingly, the error is smaller for the real data than for a single exponential case in S2(a). For example, for the largest value of τ =26.7 ns, we obtained $\bar{t}$ =23.6 ns, the error is 11.6%. Note that it was 22.5% for τ =20.0 ns for a single exponential case.

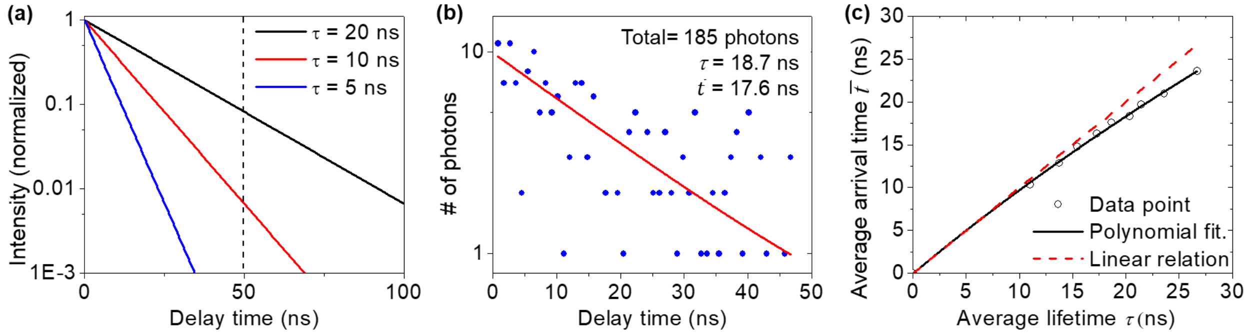

Figure S2. (a) Exponential decaying curves with their lifetimes of 20 ns (black), 10 ns (red), and 5 ns (blue). (b) The photon arrival time distribution for a selected point in FLID data. Red line is a single exponential fitting curve to derive the decay time τ. (c) Relationship between the decay time τ and the average arrival time $\bar{t}$. The black open circles are $\bar{t}$ values while the linear (red dashed, slope=1) line represents τ. The black line is a polynomial fitting curve used for the correction of the FLID data.

We should emphasize that both of τ and $\bar{t}$ have their pros and cons in estimating the average lifetime. Under our experimental circumstances, we expect that τ would be more appropriate for estimating the average lifetime for a slow-decaying case (> 10 ns) because the photons with longer arrival times than 50 ns (corresponding to 20 MHz repetition rate) are excluded in deriving of $\bar{t}$. However, for a fast-decaying case (< 10 ns) $\bar{t}$ can be more suitable because the data (decaying histograms) become more multi-exponential than a single exponential with larger contributions of shorter decays by trions.

To check the validity of our analysis to distinguish different blinking types from their FLID trajectory, the FLID histograms of PQD1-3 (in the main text) prepared by using τ instead of $\bar{t}$ is presented in Figure S3(a)-(c). Although the time axis is expanded following the error dependency (black line in Figure S2(c)), it is obvious to see that the overall features of FLID distribution does not change compared to our previous FLID histograms in the main manuscript.

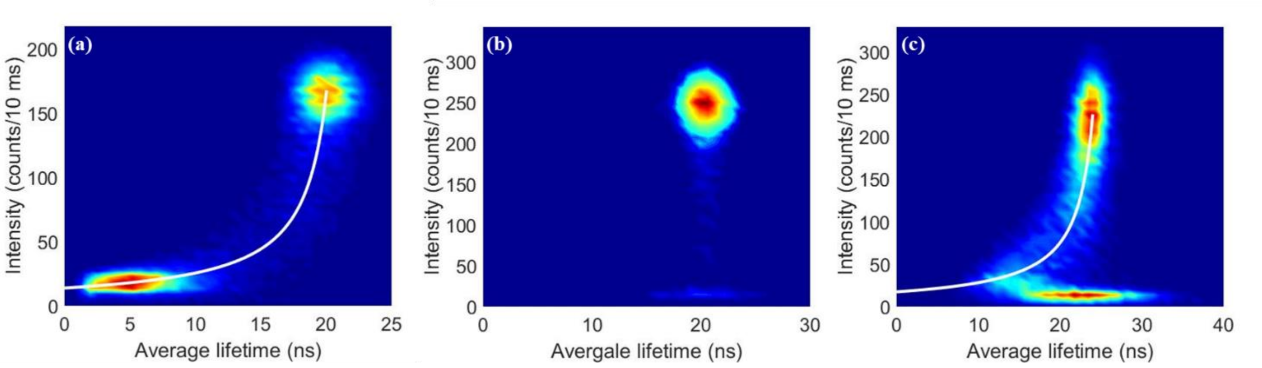

Figure S3. The FLID histograms of (a) PQD1, (b) PQD2 and (c) PQD3 (in the main text) after correction.

S3. FILD histogram of different PQDs

Type-A blinking:

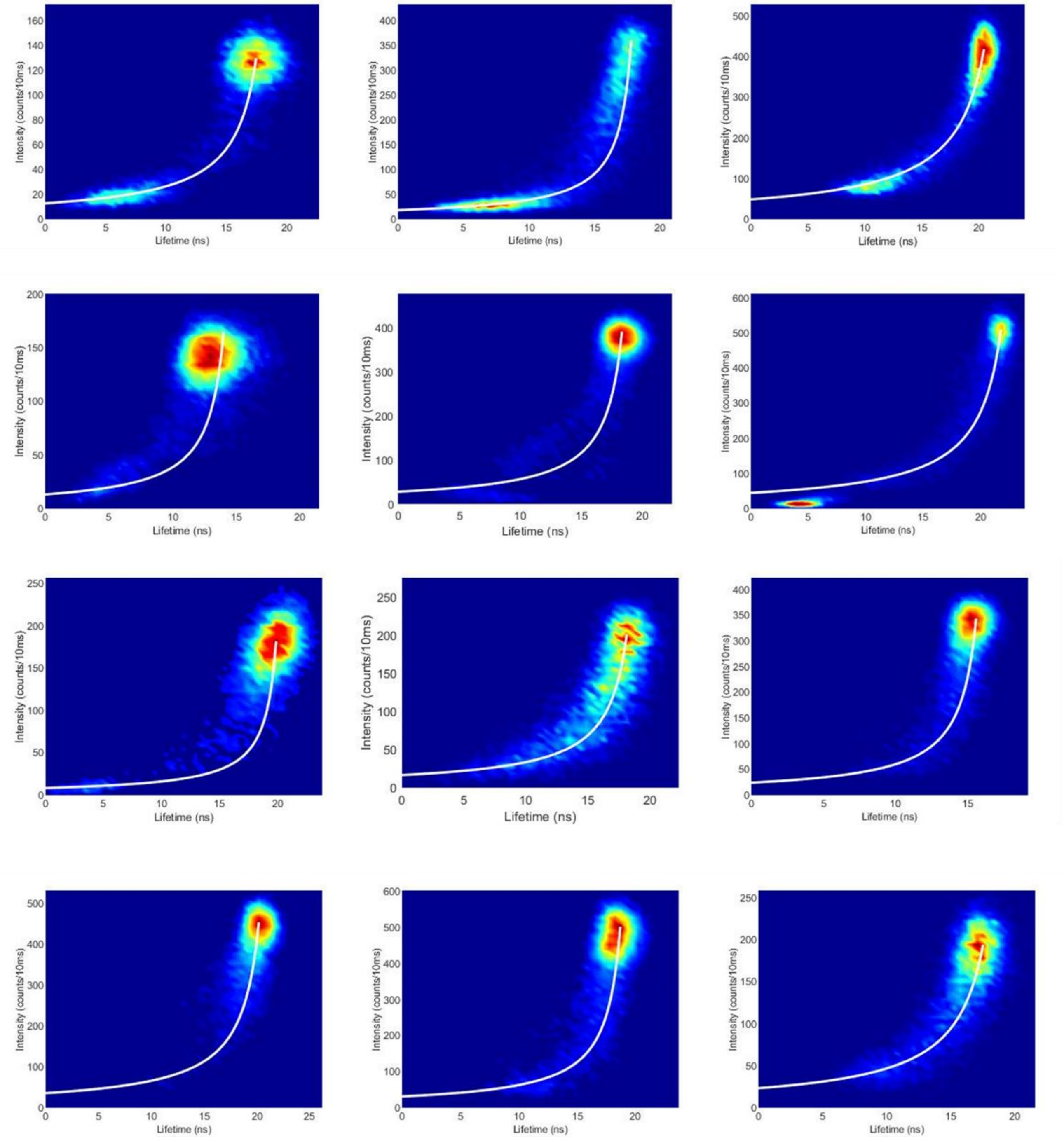

Figure S4. FLID histograms for type-A blinking.

Type-B blinking:

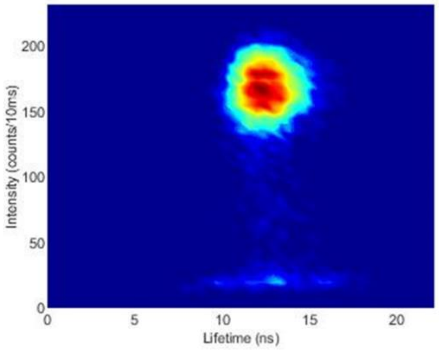

Figure S5. A FLID histogram for type-B-HC blinking.

Type-A+Type-B blinking:

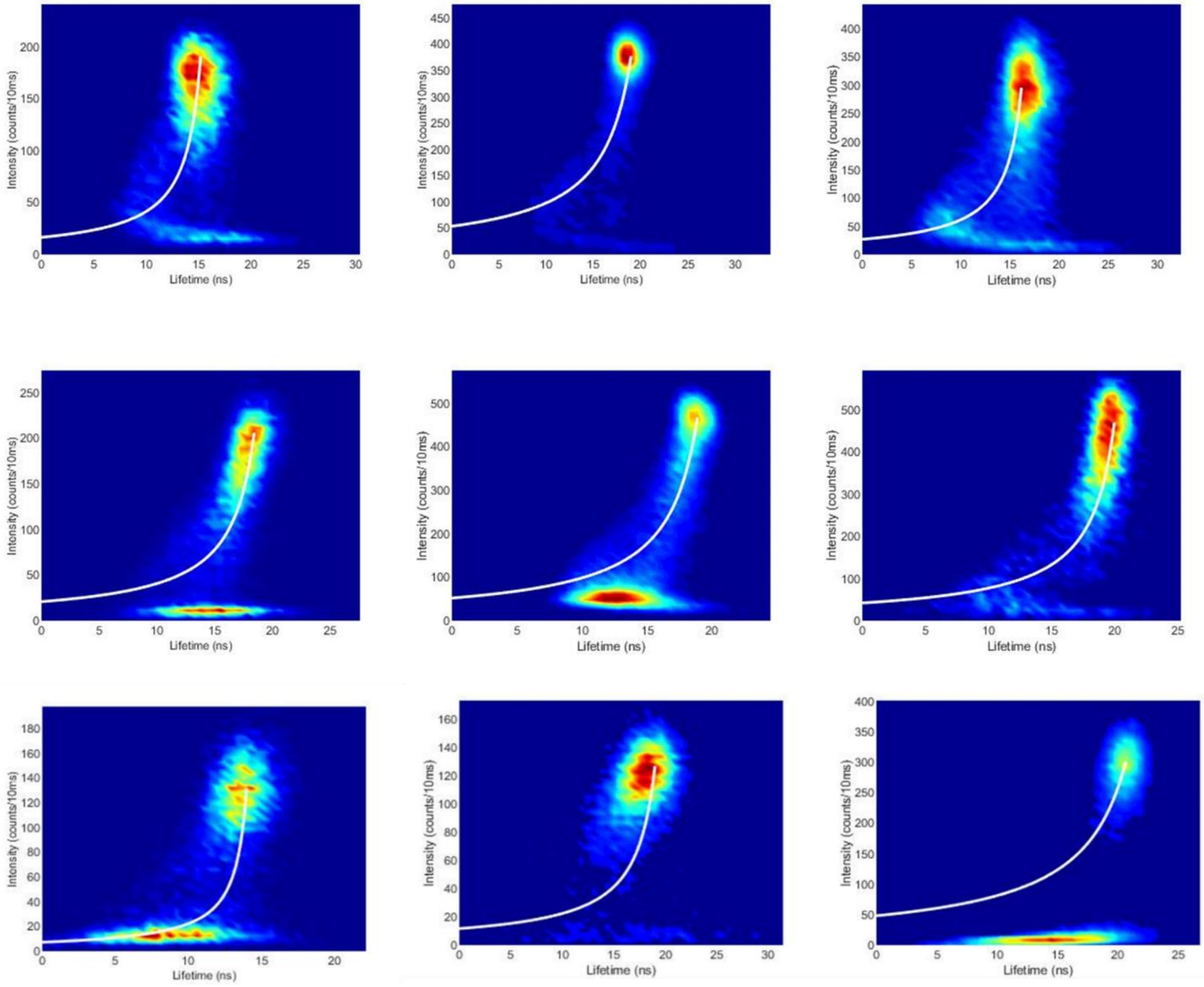

Figure S6. FLID histograms for mutual contribution of type-A and type-B-HC blinking.
